# Tyrosol induces multiple drug resistance in yeast *Saccharomyces cerevisiae*

**DOI:** 10.3389/fmicb.2023.1203243

**Published:** 2023-06-05

**Authors:** Elizaveta O. Noskova, Olga V. Markova, Dmitry A. Knorre, Kseniia V. Galkina

**Affiliations:** ^1^Faculty of Bioengineering and Bioinformatics, Lomonosov Moscow State University, Moscow, Russia; ^2^A.N. Belozersky Institute of Physico-Chemical Biology, Lomonosov Moscow State University, Moscow, Russia

**Keywords:** ABC-transporter, tyrosol, yeast, multiple drug resistance, pleiotropic drug resistance

## Abstract

In yeast, multiple (pleiotropic) drug resistance (MDR) transporters efflux xenobiotics from the cytoplasm to the environment. Additionally, upon the accumulation of xenobiotics in the cells, MDR genes are induced. At the same time, fungal cells can produce secondary metabolites with physico-chemical properties similar to MDR transporter substrates. Nitrogen limitation in yeast *Saccharomyces cerevisiae* leads to the accumulation of phenylethanol, tryptophol, and tyrosol, which are products of aromatic amino acid catabolism. In this study, we investigated whether these compounds could induce or inhibit MDR in yeast. Double deletion of *PDR1* and *PDR3* genes, which are transcription factors that upregulate the expression of PDR genes, reduced yeast resistance to high concentrations of tyrosol (4–6 g/L) but not to the other two tested aromatic alcohols. *PDR5* gene, but not other tested MDR transporter genes (*SNQ2*, *YOR1*, *PDR10*, *PDR15*) contributed to yeast resistance to tyrosol. Tyrosol inhibited the efflux of rhodamine 6G (R6G), a substrate for MDR transporters. However, preincubating yeast cells with tyrosol induced MDR, as evidenced by increased Pdr5-GFP levels and reduced yeast ability to accumulate Nile red, another fluorescent MDR-transporter substrate. Moreover, tyrosol inhibited the cytostatic effect of clotrimazole, the azole antifungal. Our results demonstrate that a natural secondary metabolite can modulate yeast MDR. We speculate that intermediates of aromatic amino acid metabolites coordinate cell metabolism and defense mechanisms against xenobiotics.

## Introduction

Microbial cells interact with a large variety of toxic organic compounds that are produced by other organisms. To prevent harm from these compounds, cells actively pump them out of the cytoplasm into the environment (Paulsen, 2003; Orelle et al., 2023). The efflux is mediated by the plasma membrane transporter proteins belonging to the ABC- and MFS-transporter families (Cannon et al., 2009). Some of these transporters show an extraordinarily broad substrate specificity and, therefore, confer multiple (pleiotropic) drug resistance (MDR) to the microbial cells. For example, the major ABC-transporter in *Saccharomyces cerevisiae*, Pdr5p, effluxes rhodamine 6G (a lipophilic cation), cycloheximide (a soluble protein synthesis inhibitor), caffeine (a naturally occurring hydrophilic alkaloid) and clotrimazole (a lipophilic azole antifungal), which significantly differ in their molecular weight and hydrophobicity (Kolaczkowski et al., 1996; Golin et al., 2007; Sürmeli et al., 2019).

Many fungal multidrug ABC-transporters, including Pdr5p, exhibit basal ATP hydrolysis activity, enabling them to hydrolyze ATP without substrate transport (Gupta et al., 2011; Orelle et al., 2023). Therefore, fungal cells repress ABC-transporter genes under normal conditions. However, when xenobiotic molecules are accumulated in the cytoplasm, the Pdr1/Pdr3 transcription factor binds them and triggers the expression of MDR genes (Thakur et al., 2008). Pdr1p and Pdr3p transcription factors control the expression of several MDR genes, including PDR5, SNQ2, YOR1, PDR10, and PDR15 (Fardeau et al., 2007; Gulshan and Moye-Rowley, 2007). Additionally, some xenobiotics induce the expression of MDR genes indirectly. For instance, PDR5 is induced by mitochondrial dysfunction (Panwar and Moye-Rowley, 2006), while endogenous oxidative stress induces FLR1, an MDR MFS-transporter gene (Chen et al., 2007; Galkina et al., 2019). Thus, yeast cells (1) sense and (2) pump out a large number of small molecular weight compounds that fall within a wide range of physico-chemical properties.

Fungal cells generate various secondary metabolites, some of which facilitate communication with microorganisms of the same or different species (Keller et al., 2005; Macheleidt et al., 2016). For instance, aromatic alcohols produced during aromatic amino acids degradation regulate pseudohyphae and biofilm formation in Saccharomyces and Candida yeasts (Chen et al., 2004; Alem et al., 2006; Chen and Fink, 2006). 2-Phenylethanol, tyrosol, and tryptophol, three aromatic alcohols, accumulate when nitrogen sources are limited, and in stationary phase. While phenylethanol and tryptophol induce filamentous growth in *S. cerevisiae*, the regulatory function of tyrosol is unclear (Chen and Fink, 2006). Whereas 2-phenylethanol does not induce PDR genes, P161S mutation in the MFS-transporter *FLR1* was shown to increase 2-phenylethanol resistance (Hacısalihoğlu et al., 2019; Holyavkin et al., 2023). Otherwise, it remains unclear whether these compounds can be transported by MDR-proteins and induce MDR gene expression.

Here we studied whether yeast secondary metabolites can be substrates or inducers of major MDR-transporter genes. We compared the inhibitory effects of three aromatic alcohols (2-phenylethanol, tyrosol, and tryptophol) on *S. cerevisiae* cells with deleted both PDR1 and PDR3 genes to those on wild-type cells. Our results indicate that the inactivation of MDR genes reduces yeast resistance to tyrosol but not to 2-phenylethanol and tryptophol. Additionally, we evaluated the ability of tyrosol to inhibit MDR-transporter fluorescent substrates’ efflux from cells and measured the Pdr5 protein’s accumulation in response to tyrosol supplementation in the medium.

## Materials and methods

### Yeast strains and growth conditions

In this study, we used *W303-1A* and *BY4741* haploid *Saccharomyces cerevisiae* strains and its derivative mutants. *Δpdr1Δpdr3* and *W303-1A KanMX4* control strain were obtained previously (Galkina et al., 2018). *BY4741, Δpdr5, Δsnq2, Δyor1, Δpdr10, Δpdr15* strains were from the yeast deletion collection (Giaever et al., 2002); *Pdr5-GFP* strain was from yeast GFP collection (Huh et al., 2003). To grow yeast strains we used a standard rich medium Yeast Peptone Dextrose (YPD) containing 2% of D-glucose (Helicon, Cat.No.H-0401-0.5) as the carbon source (Sherman, 2002). In order to analyze growth rates we inoculated cells into 96-well microplates (100 μL) and incubated them at 30°C. We used the SpectroStar Nano (BMG Labtech GmbH) microplate reader. The optical density at 550 nm (OD_550_) was assessed every 5 min for the entire duration of the experiment. In order to calculate a specific growth rate μ_max_ we calculated the slope of Log2(OD_550_) in a time window of 50 min during the course of the experiment (μ_max_ values are inversely proportional to the duplication time).

We used peptone (DiaM, Cat.No. HYP-A.5000) and yeast extract (DiaM, Cat.No. 0207/0-PW-L.5000) from BioSpringer, agar (Cat.No. 1923.5000) from DiaM, tyrosol (Cat.No. 27600) and tryptophol (Cat.No.28404) from Cayman Chemical Company. 2-phenylethanol (Serva) was gifted by Prof. Yaguzhinsky L.S. Clotrimazole (Cat.No. C6019-5G) was from Sigma.

### Rhodamine 6G efflux

To evaluate the activity of MDR-transporters, we quantified the efflux of the fluorescent MDR-transporter substrate rhodamine 6G (Sigma, Cat.No. R4127; Kolaczkowski et al., 1996) using a modified protocol from our previous work (Knorre et al., 2014). To load R6G into yeast cells, we induced energy deprivation by incubating exponentially grown yeast cells (~10^7^ cells/mL) in phosphate buffer saline (PBS, Gibco, Cat.No. 18912-014) containing 2-deoxyglucose (5 mM, Chem-Impex Int’l Inc., Cat.No. 21916), NaN_3_ (10 mM, Molecula, Cat.No. 31803515-0.1), and R6G (10 μM). Next, we incubated the cell suspension for 2 h on a rotary shaker (250 rpm) before removing the inhibitors and R6G from the energy-deprived cells by subjecting them to two cycles of centrifugation (1,125 g)/resuspension in PBS. The efflux of R6G was measured with a FluoroMax-3 fluorometer, with an excitation wavelength of 480 nm and an emission wavelength of 560 nm. We induced the R6G efflux by adding 1% glucose, and the cell density in the fluorometric cuvette was 10^6^ cells/mL.

### Staining of yeast cells with propidium iodide

To estimate the number of dead cells we stained them 10 min with 1 μg/mL propidium iodide (PI, Sigma, Cat.No. P4864-10ML) in phosphate buffer saline. Then we measured PI accumulation using flow cytometry with an excitation wavelength of 488 nm and an emission filter of 780/60 nm. We analyzed the level of fluorescence in at least 10,000 events (cells) in each sample.

### Accumulation of Nile red in yeast cells

To assess the accumulation of Nile red (Invitrogen, Cat.No. N1142), a fluorescent substrate of MDR-transporters, we used W303-1A wild type yeast cells in the exponential growth phase. We supplemented the cells with Nile red (3.5 μM) and incubated them for 10 min. Additionally, we investigated the effect of preincubation by supplementing the aromatic alcohols an hour before the addition of Nile red. The level of Nile red accumulation was measured using a CytoFlex flow cytometer (Beckman-Coulter) with an excitation wavelength of 488 nm. The accumulation of Nile red was measured using an emission filter of 585/42 nm. We analyzed the level of fluorescence in at least 10,000 events (cells) in each sample.

### Analysis of Pdr5-GFP levels in yeast cells

We measured the concentration of Pdr5-GFP in yeast cells using flow cytometry and fluorescence microscopy. Flow cytometry: excitation wavelength of 488 nm, Pdr5-GFP fluorescence was measured using 525/40 nm emission filter. To check the intracellular localization of Pdr5-GFP we used the fluorescence microscope Olympus BX41 with the U-MNIBA3 (excitation wavelength 470–495 nm; beamsplitter filter 505 nm; emission 510–550 nm) filter set.

### Statistics and data visualization

In this study, we used the non-parametric Wilcoxon rank sum exact test to determine whether two samples were derived from the same population. In the figures, we provided individual data points where possible, representing the results of biologically independent experiments. We performed data visualization using R and the tidyverse library ggplot2 (Wickham, 2016).

## Results

**Figure 1:**
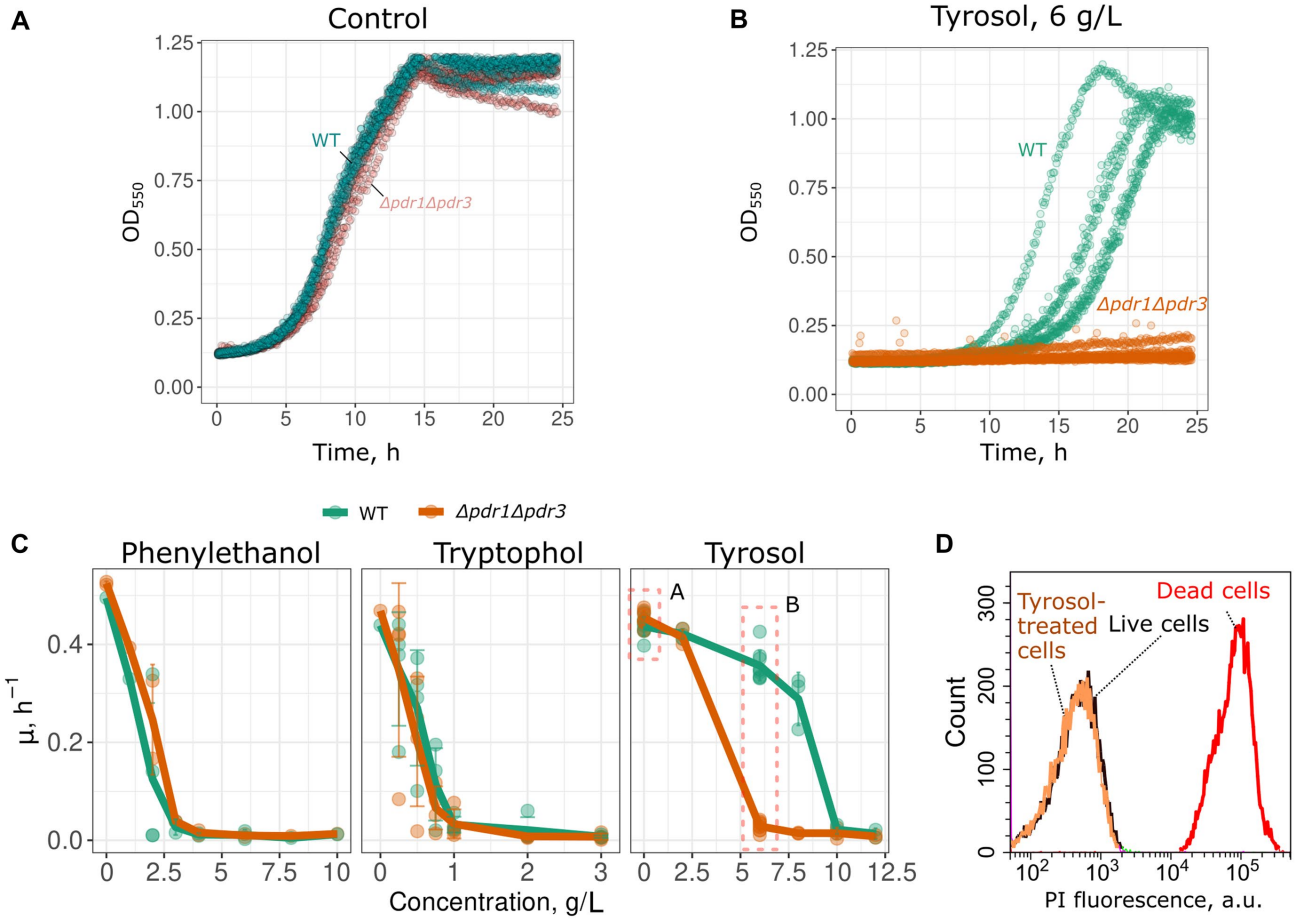
Double deletion of *PDR1* and *PDR3* genes decreases growth rate in the presence of tyrosol. Representative growth curves of WT (*W303-1A KanMX4*) and *Δpdr1Δpdr3* strains in the control conditions **(A)** and in the presence of tyrosol, 6 g/L **(B)**. *p* < 0.05 according to Wilcoxon rank sum exact test for the comparison of growth rates of WT and *Δpdr1Δpdr3* strains. **(C)** Growth rates (μ_max_) in the presence of 2-phenylethanol, tryptophol and tyrosol. **(D)** WT yeast cells incubated with tyrosol (8 g/L, 1 h of incubation) do not accumulate propidium iodide (PI). Yeast control cells untreated with tyrosol and yeast cells killed with 70% (v/v) ethanol are shown as the negative and the positive controls.

**Figure 2:**
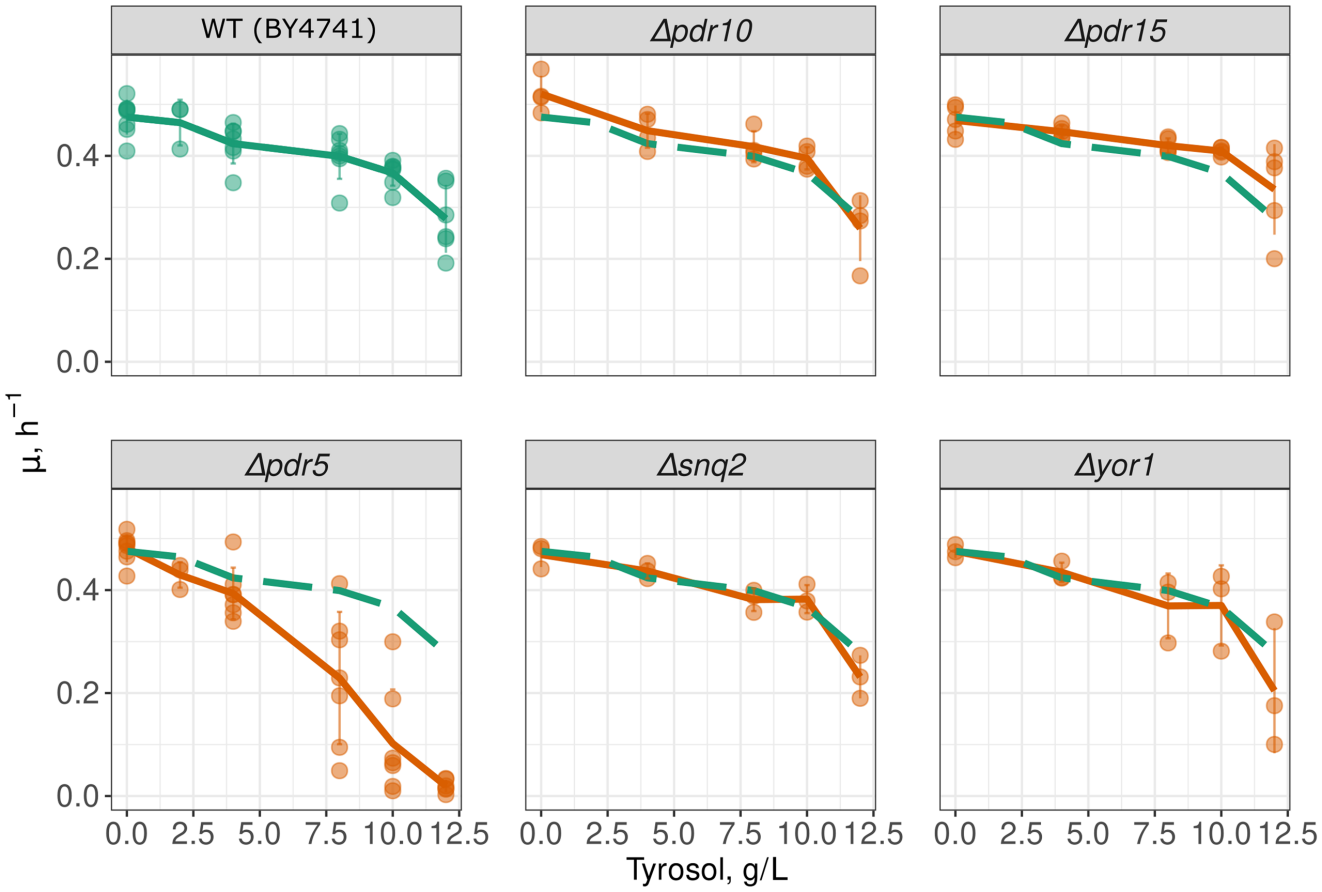
*PDR5* deletion aggravates the inhibitory effect of tyrosol on yeast growth. Growth rates (μ_max_) of yeast strains with deleted MDR genes; the growth rate of control parental strain WT (*BY4741*) is shown on all facets as the green long-dash line.

To determine whether MDR genes contribute to the resistance of yeast cells to 2-phenylethanol, tyrosol, and tryptophol, we compared growth rate of a wild-type and MDR-deficient (*Δpdr1Δpdr3*) strains in the presence of these compounds. We chose the *Δpdr1Δpdr3* strain for this analysis because it exhibits no growth defects under normal conditions (Figure 1A). Double deletion of *PDR1* and *PDR3* did not increase yeast sensitivity to 2-phenylethanol and tryptophol. At the same time, the growth of the *Δpdr1Δpdr3* strain was completely abolished by tyrosol at a concentration of 6–8 g/L, whereas the wild-type strain was only slightly inhibited (Figures 1B,C). At the same time, tyrosol did not induce the death of yeast cells: incubation of wild type yeast cells did not induce accumulation of propidium iodide in yeast cells (Figure 1D). To identify the specific target of these transcription factors that contribute to tyrosol tolerance, we tested the growth rate of several yeast knockout strains lacking one of the MDR genes. In these experiments we used strains from yeast knockout collection and, therefore, took *BY4741* strain as the parental control. Our results showed that the deletion of the *PDR5* gene, but not other MDR genes: *SNQ2*, *YOR1*, *PDR10*, or *PDR15*, decreased yeast cell growth in the presence of tyrosol (Figure 2).

**Figure 3:**
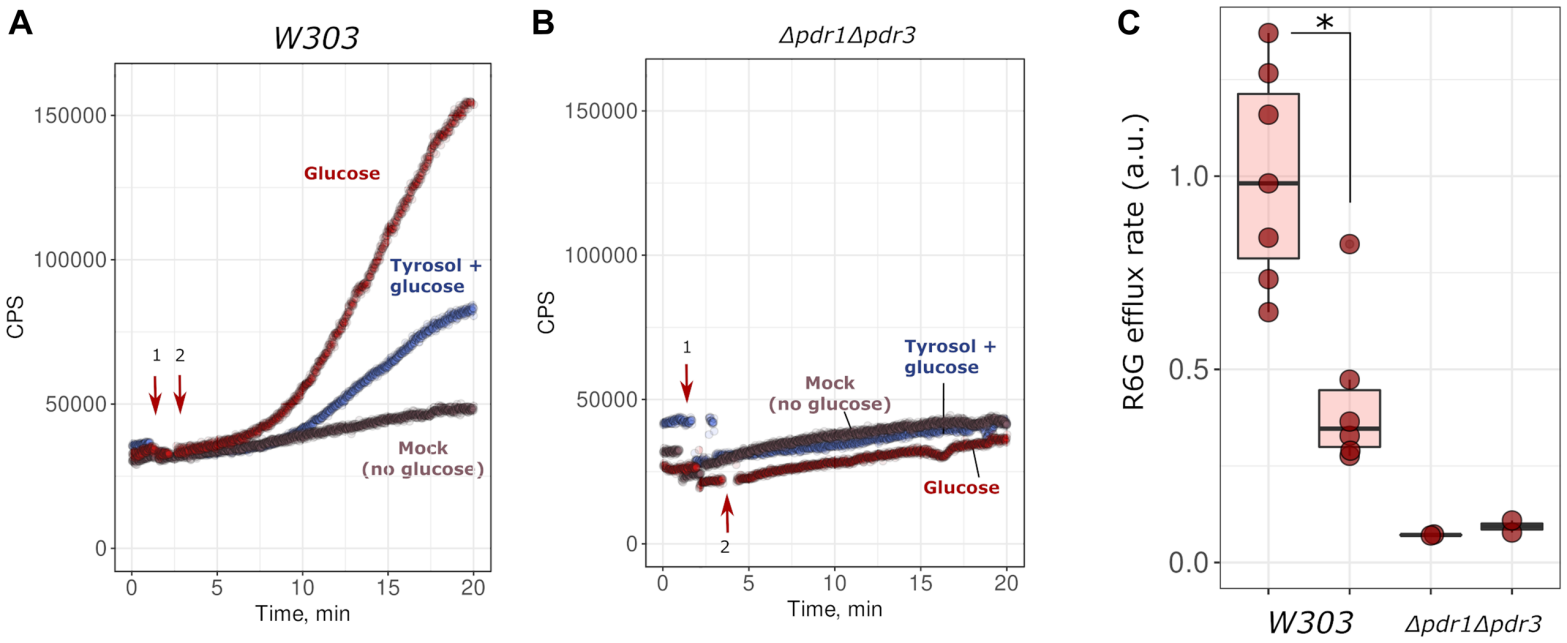
Tyrosol inhibits glucose-stimulated rhodamine 6G efflux from yeast cells. We monitored rhodamine 6G efflux from yeast cells by the increase in fluorescence (excitation wavelength λ = 480 nm, emission wavelength λ = 560 nm; see Material and methods sections for more details). Wild type *W303-1A KanMX4* **(A)** or *Δpdr1Δpdr3* **(B)** yeast cells were supplemented first with tyrosol or solvent (1, indicated by arrow), and then with glucose (2, indicated by arrow). **(C)** Quantification of the results from three separate day experiments. *corresponds to *p* < 0.05 according to Wilcoxon rank sum exact test.

Given that the inactivation of MDR genes reduced the resistance of yeast to tyrosol, we hypothesized that tyrosol could be a substrate of one of the MDR-transporters, competing with other MDR carrier substrates and reducing their release rate from cells. To test this, we measured glucose-induced R6G efflux from yeast cells. R6G fluorescence is quenched at high concentrations. Upon the addition of glucose, cells synthesize ATP and pump-out R6G with MDR-transporters, resulting in an increase in the integral fluorescence of the suspension. The supplementation of glucose to energy-deprived yeast cells induced R6G efflux to the incubation medium (Figure 3A). This effect was negligible in the *Δpdr1Δpdr3* strain suggesting negligible efflux of R6G (Figure 3B). We found that high concentration of tyrosol (4 g/L) inhibited the R6G efflux (Figures 3A,C). These results suggest that tyrosol is a substrate of the MDR-transporters and competes with other carrier substrates for efflux from the cells.

**Figure 4:**
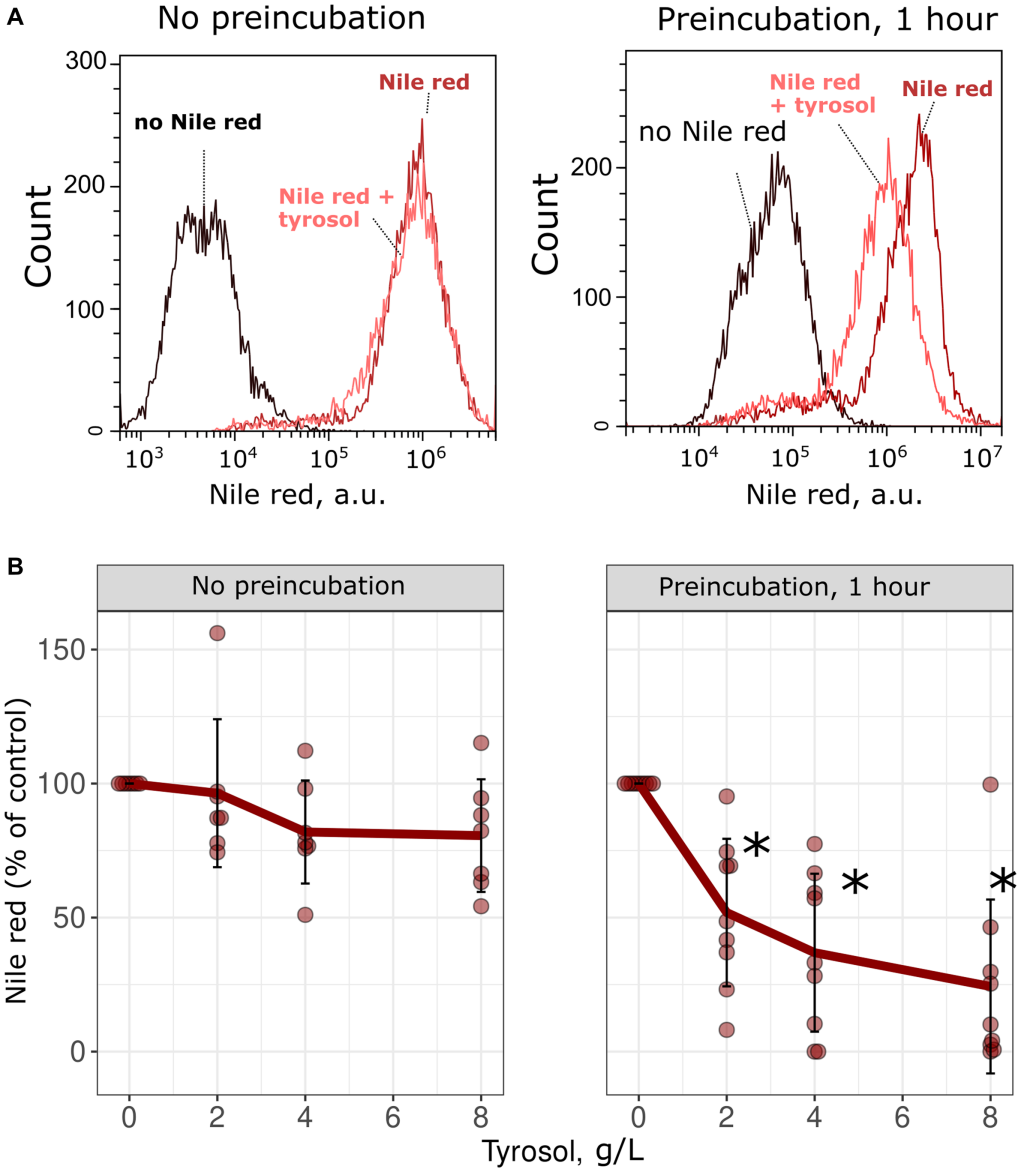
Tyrosol decreases the concentration of MDR-transporter substrate Nile Red in yeast cells. **(A)** Supplementation of tyrosol (4 g/L) to the yeast suspension does not change the accumulation levels of Nile red in wild type *W303-1A KanMX4* yeast cells (left). One hour preincubation of yeast cells with tyrosol (4 g/L) inhibits Nile red accumulation in yeast cells (right). **(B)** Quantification of the results for different concentrations of the Nile red. Median Nile red level in untreated cells was set as 100%, mean autofluorescence signal was subtracted. *corresponds to *p* < 0.05 according to Wilcoxon rank sum exact test.

**Figure 5:**
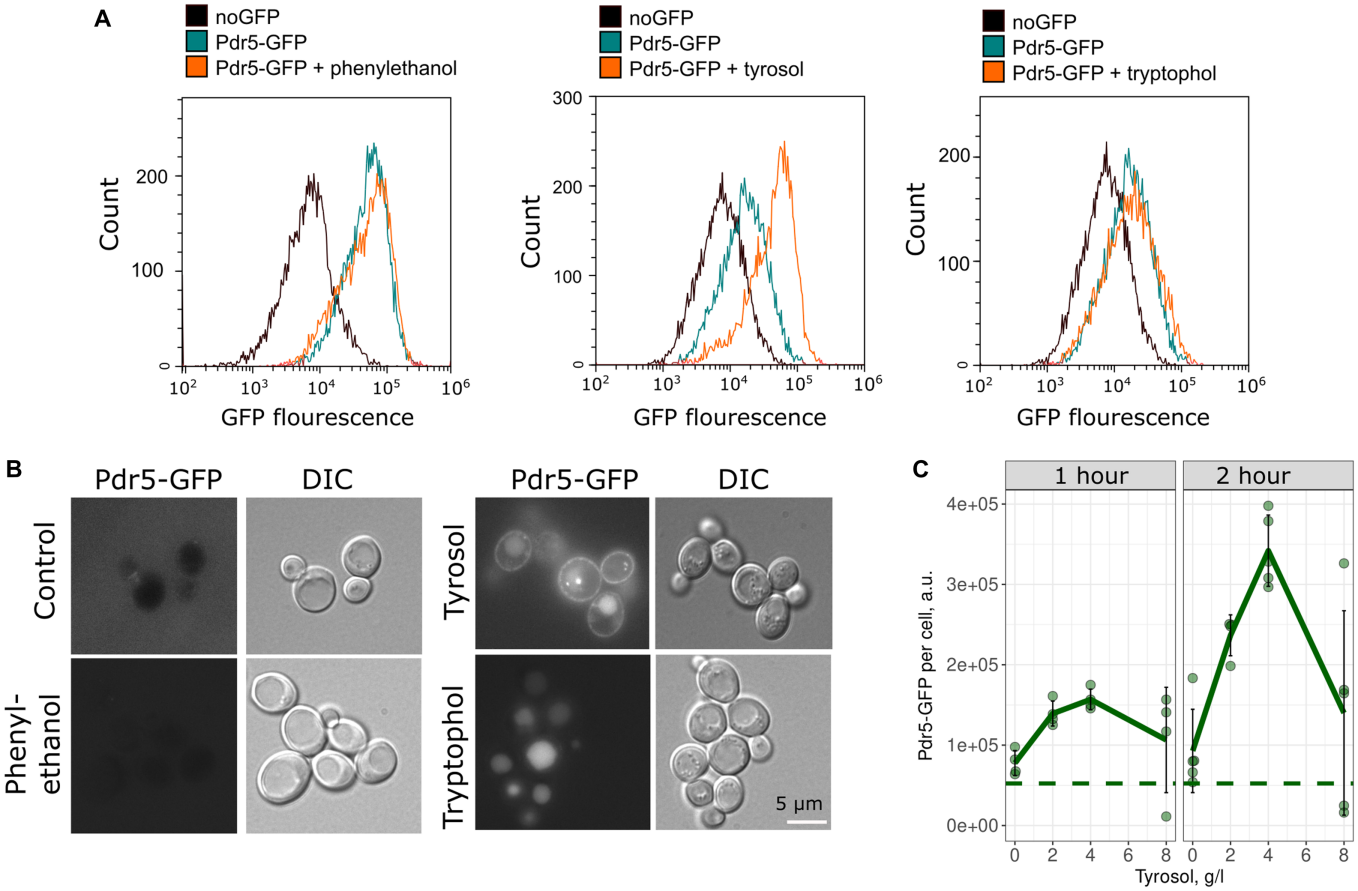
Tyrosol induces accumulation of Pdr5-GFP in the yeast plasma membrane. **(A)** Flow cytometry analysis was conducted to measure the levels of Pdr5-GFP in yeast cells treated with 2-phenylethanol (1 g/L), tyrosol (4 g/L), and tryptophol (0.75 g/L) for 1 h. **(B)** The localization of the GFP signal in yeast cells treated with 2-phenylethanol (2 g/L), tyrosol (4 g/L), and tryptophol (1 g/L); **(C)** Quantification of the flow cytometry results for yeast cells treated with tyrosol (1 and 2 h), dots represent mean Pdr5-GFP fluorescence from a separate experiment (10,000 events, *n* = 4). The dashed line represents the mean fluorescence of Pdr5-GFP before the addition of aromatic alcohols.

To confirm the inhibitory effect of tyrosol on MDR-transporters, we analyzed its effect on the accumulation of Nile red, another fluorescent MDR substrate. We measured the accumulation levels of Nile red in yeast cells using flow cytometry. Surprisingly, supplementation of yeast cells with tyrosol did not increase Nile red levels (Figure 4). Moreover, preincubation of yeast cells with tyrosol for 1 h prior to the addition of Nile red significantly reduced the Nile red accumulation levels in the cells (Figure 4). We then investigated whether the decrease in Nile red concentration in cells is linked to upregulation of MDR-transporter genes by analyzing the levels of the major yeast MDR-transporter, Pdr5, fused with GFP. Our results showed that tyrosol, but not tryptophol or phenylethanol, increased Pdr5-GFP levels in cells (Figure 5). For this experiment, we chose concentrations of aromatic alcohols that inhibited but did not completely stop the growth of yeast cells. Importantly, we found that tyrosol increases Pdr5-GFP levels in plasma membranes (Figure 5B).

**Figure 6:**
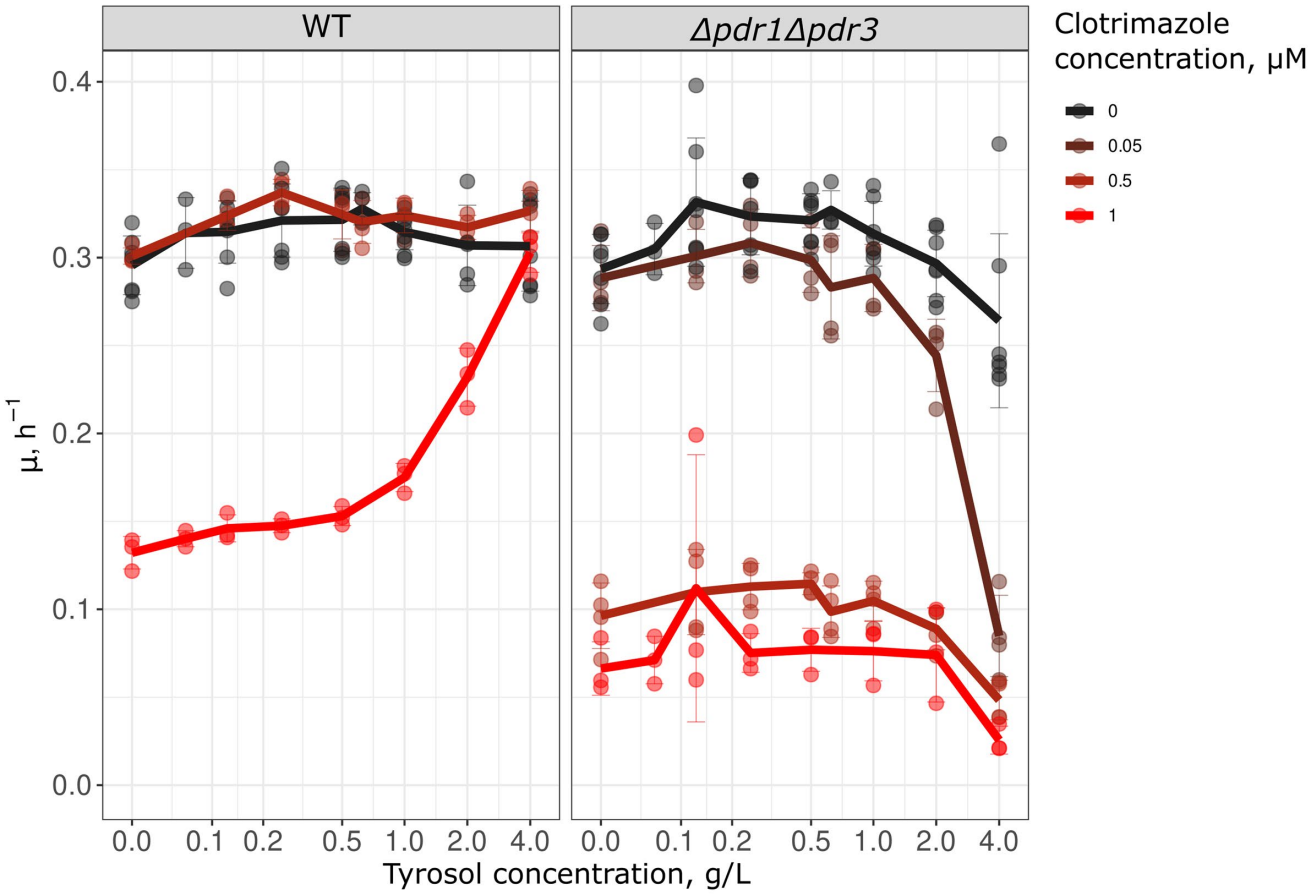
Tyrosol cancels the growth inhibition effect of clotrimazole. We analyzed the growth rate (μ_max_) in WT (*W303-1A KanMX4*) and *Δpdr1Δpdr3* strains to determine the effect of tyrosol on the cytostatic effect of clotrimazole. The lines on the graph represent the average μmax of 3–6 independent experiments.

Since preincubation with tyrosol increased Pdr5 levels and inhibited Nile red accumulation, we hypothesized that tyrosol could prevent the adverse effects of antifungals. To test this, we measured the growth kinetics of yeast cells treated with the combinations of different concentrations of tyrosol and clotrimazole, an azole antifungal that inhibits ergosterol biosynthesis. We found that supplementation of yeast cells with 0.5–4 g/L tyrosol rescued yeast growth inhibited by 1 μM clotrimazole (Figure 6). To verify that this effect depends on MDR genes we analyzed the effect of tyrosol and clotrimazole mixtures on *Δpdr1Δpdr3* cells growth rates. Given that *Δpdr1Δpdr3* strain is much more sensitive to clotrimazole, we also tested the effect of tyrosol on yeast treated with lower concentrations of clotrimazole (0.5 and 0.05 μM). In contrast to wild type strain, at high concentrations (2–4 g/L), tyrosol enhanced the cytostatic effect of 0.05 μM clotrimazole in the *Δpdr1Δpdr3* strain (Figure 6).

## Discussion

In this study, we have observed that aromatic alcohols exhibit inhibitory effects on the growth of *S. cerevisiae* in high concentrations, reaching several grams per liter, as shown in Figure 1. Under normal conditions, yeast are incapable of producing these alcohols in such significant quantities, with growth media typically accumulating only 0.01–0.02 g/L of phenylethanol, tyrosol, and tryptophol (Zupan et al., 2013; Kunyeit et al., 2021). Meanwhile, overexpression of individual ARO genes can increase the concentration of tyrosol to approximately 0.05 g/L (Bisquert et al., 2022). By heterologous gene expression, metabolic flux can be redirected to enable yeast to synthesize tyrosol from exogenous tyrosine. This results in a tyrosol yield of around 1.2–1.4 g/L in batch cultures (Jiang et al., 2018; Guo et al., 2019). Moreover, rewiring of amino acid metabolism via metabolic engineering can increase the concentration of tyrosol to approximately 0.7 g/L in batch cultures and up to 9.9 g/L in bioreactors (Liu et al., 2021).

In our study, the concentrations of tyrosol that were found to inhibit yeast growth and induce MDR were two orders of magnitude higher than the concentration of tyrosol observed in the incubation media of stationary phase wild-type yeast cells. Hence, it is unlikely that cells can utilize tyrosol to elicit MDR in neighboring cells. Meanwhile, if the plasma membrane acts as a barrier for tyrosol diffusion, the concentration of tyrosol in the cytoplasm of the tyrosol-producing cell can surpass the concentration in the medium. The molecular structure of tyrosol, along with its hydrophobicity (logP is equal to 1.0 according to logP prediction software Molinspiration, www.molinspiration.com, Slovensky Grob, Slovakia), suggests that tyrosol can passively diffuse through the membrane. Therefore, the efflux of this compound should be of low efficiency. Indeed, Lipinsky rule of five suggests good bioavailability and membrane permeation for drugs with a LogP falling in range (− 0.5 < LogP < 5; Soliman et al., 2021). However, diffusion of tyrosol across the membrane can be rate-limited. Therefore, if tyrosol is actively effluxed out of the cells, a gradient can be maintained between tyrosol-producing cells and the environment. And vice versa, the concentration of tyrosol in yeast cells treated with exogenous tyrosol would be lower than the concentration of tyrosol in the medium. Hence, we expect that the addition of exogenous tyrosol to the cells will lead to an increase in the intracellular concentration of tyrosol within the physiological range of concentrations.

Our study revealed that double deletion of transcription factor genes *PDR1* and *PDR3*, along with the deletion of *PDR5* gene encoding major yeast ABC-transport, increased yeast sensitivity to tyrosol but not tryptophol and phenylethanol (Figures 1, 2). This result suggests that Pdr5p plays a role in the efflux of tryptophol from yeast cells. The ability of tyrosol to inhibit R6G efflux, a MDR-transporter substrate (Kolaczkowski et al., 1996), supports this assumption (Figure 3). However, supplementation of tyrosol did not inhibit the accumulation of another MDR-transporter substrate, Nile red (Figure 4). This conflicting result can be explained by the difference in substrate specificity of MDR-transporters toward R6G and Nile red. Although MDR genes exhibit broad specificity toward transporting substrates, specific transporters can be more efficient with some substrates than others. For instance, in *Candida albicans*, overexpression of *CDR1* and *MDR1* inhibits Nile red accumulation in yeast cells, whereas only overexpression of *CDR1* was effective in preventing R6G accumulation (Ivnitski-Steele et al., 2009). *CDR1* is the orthologue of *S. cerevisiae PDR5* gene, *MDR1* encoded MFS-transporter with broad substrate specificity that is orthologues to *S. cerevisiae FLR1*. Therefore, we suggest that the absence of inhibition of Nile red efflux by tyrosol can be explained if it is effluxed by an MDR-transporter that is insensitive to tyrosol and cannot efflux R6G.

Our study found that tyrosol induces the accumulation of Pdr5-GFP (Figure 5). This observation is in line with RNAseq experiments that showed that tyrosol induces MDR genes *CDR1* and *MDR1* in *Candida parapsilosis* (Jakab et al., 2019). Phenylethanol, tryptophol, and tyrosol are produced by both *S. cerevisiae* and *Candida species*, but the physiological response to aromatic alcohol differs between yeast species. So, tyrosol appears to be a quorum-sensing molecule in *Candida albicans* that induces cell morphogenesis (Chen et al., 2004). However, only phenylethanol and tyrosol induce morphological changes in *S. cerevisiae*, whereas tyrosol proved to be inefficient (Chen and Fink, 2006). It turns out that tyrosol, unlike the other two aromatic alcohols, does not have a specific target in *S. cerevisiae* and is probably perceived by cells as a xenobiotic when its concentration reaches a certain threshold.

In line with the results showing that tyrosol activates MDR, we found that tyrosol, when added simultaneously with the antifungal drug clotrimazole, is able to cancel its cytostatic activity and restore cell growth (Figure 6). Clotrimazole is an inhibitor of ergosterol biosynthesis (Kathiravan et al., 2012) and the substrate of Pdr5 ABC-transporter (Golin et al., 2003). We assume that tyrosol causes the production of MDR pumps before clotrimazole depletes ergosterol in the cell, inhibiting proliferation.

To summarize, in this study we found that tyrosol, added in high concentration, induces MDR and is likely a substrate of MDR-transporters. Thus, the accumulation of tyrosol in tyrosol-producing yeast cells can be an endogenous trigger upregulating drug resistance under conditions of aromatic amino acids metabolism. Our findings show that a secondary metabolite can be a signaling molecule that activates MDR in yeast cells, thus coordinating cellular metabolism and the cell’s response to stress.

## Data availability statement

The original contributions presented in the study are included in the article, further inquiries can be directed to the corresponding author.

## Author contributions

KG and DK designed and supervised the research and wrote the text and prepared the illustrations. KG obtained funding. KG and EN conducted the experiments. OM participated in rhodamine 6G efflux experiments. All authors contributed to the article and approved the submitted version.

## Funding

The study was supported by Russian Science Foundation No: 22-24-00406.

## Conflict of interest

The authors declare that the research was conducted in the absence of any commercial or financial relationships that could be construed as a potential conflict of interest.

## Publisher’s note

All claims expressed in this article are solely those of the authors and do not necessarily represent those of their affiliated organizations, or those of the publisher, the editors and the reviewers. Any product that may be evaluated in this article, or claim that may be made by its manufacturer, is not guaranteed or endorsed by the publisher.
